# Identifying Active Substances and the Pharmacological Mechanism of *Houttuynia cordata* Thunb. in Treating Radiation-Induced Lung Injury Based on Network Pharmacology and Molecular Docking Verification

**DOI:** 10.1155/2022/3776340

**Published:** 2022-03-22

**Authors:** Gui-Hua Lai, Fei Wang, Duo-Rui Nie, Shu-Jun Lei, Zhuo-Jun Wu, Jian-Xiong Cao

**Affiliations:** ^1^Graduate School, Hunan University of Chinese Medicine, Changsha, Hunan 410208, China; ^2^School of Continuing Education, Hunan University of Chinese Medicine, Changsha, Hunan 410208, China

## Abstract

**Background:**

*Houttuynia cordata* Thunb. is a traditional Chinese herb widely used mainly because of the pharmacological effects related to heat clearance and detoxification. Emerging clinical evidence indicates that the efficacy of *Houttuynia cordata* Thunb. on RILI is upstanding. Nevertheless, its underlying therapeutic mechanism remains unclear and warrants further elucidation.

**Methods:**

The major active components and corresponding targets of *Houttuynia cordata* Thunb. were retrieved from the traditional Chinese medicine system pharmacology database (TCMSP) and literature review. The related targets of RILI were retrieved from the GeneCards database. Common targets among the active compounds and diseases were identified through Venn diagram analysis. Cytoscape was employed to construct and visualize the network relationship among the drug, active compounds, targets, and disease. The protein interaction network (PPI) was constructed by STRING. The reliability (the binding affinity) of the core targets and active compounds was verified by molecular docking.

**Results:**

A search of the TCMSP database and related literature revealed 12 active compounds of *Houttuynia cordata* Thunb. against RILI. The core active compounds included quercetin, kaempferol, hyperoside, and rutin. Hub nodes including TP53, VEGFA, JUN, TNF, and IL-6 were identified in the PPI network. The GO categories were classified into three functional categories: 112 biological processes, 9 molecular functions, and 32 cellular components of the active compounds of *Houttuynia cordata* Thunb. The KEGG pathway enrichment analysis demonstrated the enrichment of target genes in several key cancer-related signaling pathways, including the cancer pathways, TNF signaling pathway, PI3K-Akt signaling pathway, and HIF-1 signaling pathway. Molecular docking analysis validated the effective binding capacity of the main active compounds with the core targets.

**Conclusion:**

The main active components of *Houttuynia cordata* Thunb. have a potential pharmacological effect against RILI via the cancer pathways, TNF signaling pathway, and PI3K-Akt signaling pathway.

## 1. Introduction

Radiation-induced lung injury (RILI) is the most common and serious complication of radiotherapy for breast cancer, lung cancer, and esophageal cancer. Compelling evidence shows that the incidence of asymptomatic RILI is as high as 43%, whereas the incidence of clinically diagnosed asymptomatic RILI is 5–15% [1, 2]. RILI significantly influences the radiation dose to the area targeted by thoracic tumor radiotherapy. The resultant reduction in the radiotherapy effect and the local control rate of the tumor interrupts radiotherapy in mild cases and may cause death in severe cases due to lung failure. This consequently impacts the quality of life and long-term survival of tumor patients [3]. Although the mechanism of RILI is not fully understood, researchers suggest a significant correlation of RILI with oxidative cell damage, inflammatory cytokine release, and immune imbalance [4, 5]. Clinical treatment is mainly based on glucocorticoids, radiation protection agents, including amphotericin, and broad-spectrum cytoprotective agents such as amifostine [6]. However, the efficacy of this approach is unsatisfactory due to the short duration of drug action and adverse drug reactions [7]. In this view, strategies for administering effective and safe treatment for RILI remain an urgent clinical concern today.

Mounting clinical evidence has demonstrated a certain degree of clinical efficacy and upstanding safety with the use of traditional Chinese medicine (TCM) to manage RILI [8]. TCM believes that the basic pathogenesis of RILI is fire, heat, toxicity, and dryness. On the other hand, the basic treatment mechanisms of RILI involve heat clearance, detoxification, nourishment of yin, and blood invigoration [9]. *Houttuynia cordata* Thunb., one of the oldest traditional Chinese herbs in China, with a history of clinical application for more than a thousand years, has been included in the Chinese Pharmacopoeia since 1963. *Houttuynia cordata* Thunb., as a representative drug in the category of purging heat and detoxification, is a genus of Houttuynia in the family Sambucus, characterized by a pungent taste and a slightly cold nature, and belongs to the lung meridian. It is frequently clinically used in combination with other herbal medicines to manage lung-phase diseases, including asthma, pneumonia, and bronchitis [10, 11]. Modern pharmacological studies have not only shown various pharmacological effects of *Houttuynia cordata* Thunb., including antiinflammatory and antioxidant but also demonstrated its inhibitory effects against pulmonary fibrosis and tumors [12–14]. Several literature reports show the antiradioactive effect of *Houttuynia cordata* Thunb. on lung injury, but its specific active compounds and molecular mechanisms against RILI remain elusive.

Network Pharmacology is pivotal in the study of the possible mechanisms of action of drugs in the following aspects: (i) mining drug and disease data in public databases; (ii) analysis of the active compounds of drugs and target genes of related diseases; (iii) exploring the possible mechanisms of action of drugs to reveal the pharmacological effects of multicomponent, multipathway, and multitarget therapeutic diseases of TCM [15]. Moreover, molecular docking is based on software that predicts binding stability between the chemical components of TCM and related targets to a certain extent, which can further verify and support the network pharmacology results [16].

**Figure 1:**
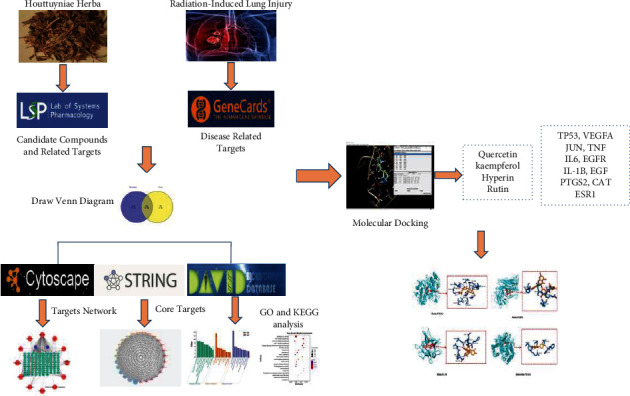
The detailed flowchart of the study design.

Therefore, this study aimed to use a comprehensive network pharmacology-based approach to investigate the potential effective components and molecular mechanisms of *Houttuynia cordata* Thunb. in treating RILI and to use molecular docking for reverse verification. The findings provide objective evidence for preliminary elucidation of the pharmacological effects and molecular mechanisms of *Houttuynia cordata* Thunb. in treating RILI. The main framework of this research is shown in Figure 1.

## 2. Materials and Methods

### 2.1. Materials

The databases used in this study include the following: CNKI (https://www.cnki.Net/), PubMed (https://www.Pubmed.ncbi.nlm.nih.gov/), TCMSP (https://tcmspw.com/tcmsp.php) [17], PubChem (https://pubchem.ncbi.nlm.nih.gov/) [18], ECTM (http://www.tcmip.cn/ETCM/index.php/Home/) [19], GeneCards (https://www.genecards.org/) [20], Venn (https://bioinfogp.cnb.csic.es/tools/venny/), Uniprot (https://www.uniprot.org/), String (https://string-db.org/) [21], David (https://david.ncifcrf.Gov) [22], OmicShare (https://www.omicshare.com/), and RCSB PDB (https://www.rcsb.org/) [23], and the analysis software used in this study include Cytoscape 3.7.1 [24], Open Babel 2.4.2, Auto Dock 4.2.6 [25], and PyMOL (https://pymol.org/2/).

### 2.2. Methods

#### 2.2.1. Components and Related Targets of *Houttuynia cordata* Thunb

The chemical constituents of *Houttuynia cordata* Thunb. were obtained from the TCMSP database and literature. According to the pharmacokinetic characteristics, oral bioavailability (OB) ≥30% and drug similarity (DL) ≥0.18 were applied as the screening conditions to obtain the main active compounds [26, 27]. Subsequently, the PubChem CIDs of the above active compounds were searched in the PubChem database. The corresponding targets of the active compounds of drug were searched in TCMSP and ETCM databases. Finally, the target name was unified and standardized via the Uniprot database and assigned a gene name.

#### 2.2.2. Intersection of Drug Target Genes with Disease Target Genes

The GeneCards database was searched using the keyword “radiation-induced lung injury” to collect the target genes related to RILI. This was followed by mapping the RILI-related genes to the drug target genes and screening the common targets to obtain the key targets of *Houttuynia cordata* Thunb. in RILI treatment. Next, the key targets of RILI were mapped to RILI-related genes and the common targets were screened to identify the key targets for RILI treatment. The web-based online mapping software Venny 2.1 was used to construct a Venn diagram of RILI mapping targets.

#### 2.2.3. Drugs, Compounds, Targets, and Disease Networks

The active compounds and related targets of *Houttuynia cordata* Thunb. were input into Cytoscape software to construct a “Drug-Compounds-Targets-Disease” topological network. The important nodes in the network were selected based on the degrees of freedom of the topological structure of the network nodes. A higher degree value of a node implied that it is more important in the network. Such an observation indicated that the node is involved in more biological functions and, therefore, is more biologically important.

#### 2.2.4. Construction and Analysis of the PPI Network

The online STRING database was used to construct a PPI network for *Houttuynia cordata* Thunb.-based treatment of RILI with the species restriction “Homospiece” and an Interaction Score >0.7 as the screening criterion. Topological analysis of the PPI network was conducted by the CytoHubba plug-in in Cytoscape (*P* < 0.01). The hub targets of *Houttuynia cordata* Thunb. for RILI treatment were further screened.

#### 2.2.5. GO Enrichment Analysis and KEGG Pathway Analysis

The DAVID database was used to perform GO functional enrichment analysis on the core targets of *Houttuynia cordata* Thunb. against RILI to understand the process of target action. Also, KEGG pathway analysis was performed to evaluate the distribution of targets in the pathway, with *P* < 0.05 and FDR < 0.05 as the screening criteria. Results were visualized via the OmicShare platform.

#### 2.2.6. Molecular Docking between Core Targets and Key Compounds

The core targets in the PPI network were molecularly docked to the four main active compounds of *Houttuynia cordata* Thunb. using AutoDock Vina software. First, the 2D structures of the core compounds were downloaded from the PubChem database and optimized in Open Babel. Subsequently, the PDB ID and 3D structures of the target proteins were downloaded from the RCSB PDB. The original PDB file was processed with PyMOL software to remove water and original ligands. Using the AutoDock Tools, hydrogen, and charge were added to the protein structure. According to the position of the ligand, the coordinates of the protein active site were determined by AutoDock Vina for molecular docking studies. Lastly, the results were visualized in PyMOL.

## 3. Results

### 3.1. Chemical Components and Putative Targets of *Houttuynia cordata* Thunb

A search in the TCMSP database yielded 50 active compounds of *Houttuynia cordata* Thunb. by screening, 7 active compounds were screened with OB ≥ 30% and DL ≥ 0.18 as the qualifying conditions. 95 targets were obtained. A literature search revealed the main pharmacological components of *Houttuynia cordata* Thunb., including methylnonane, cichorin, rutin, chlorogenic acid, and chrysin [28, 29]. Also, 35 targets were identified in the ECTM database. Active compounds from literature sources were screened according to ADME. Notably, although the OB and DL values did not meet the screening criteria, they were in the analysis because of their effective clinical and pharmacological effects. As such, a total of 12 active compounds and 101 targets of *Houttuynia cordata* Thunb. were obtained (Table 1).

### 3.2. Intersection of Drug Target Genes and Disease Target Genes

**Figure 2:**
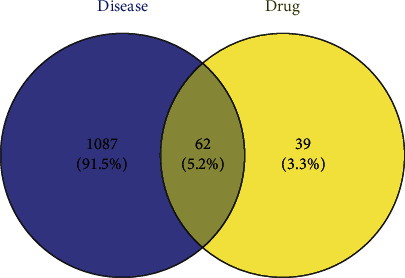
The overlapping genes of RILI and *Houttuynia cordata* Thunb.

Of the 1251 RILI-related target genes identified in GeneCards, we remained with 1149 genes after deleting those that did not correspond to the UniProt number. Next, 62 common targets were obtained by intersecting 101 targets of the active compounds of *Houttuynia cordata* Thunb. and 1149 RILI-related targets (Figure 2).

### 3.3. Drugs, Compounds, Targets, and Disease Networks

**Figure 3:**
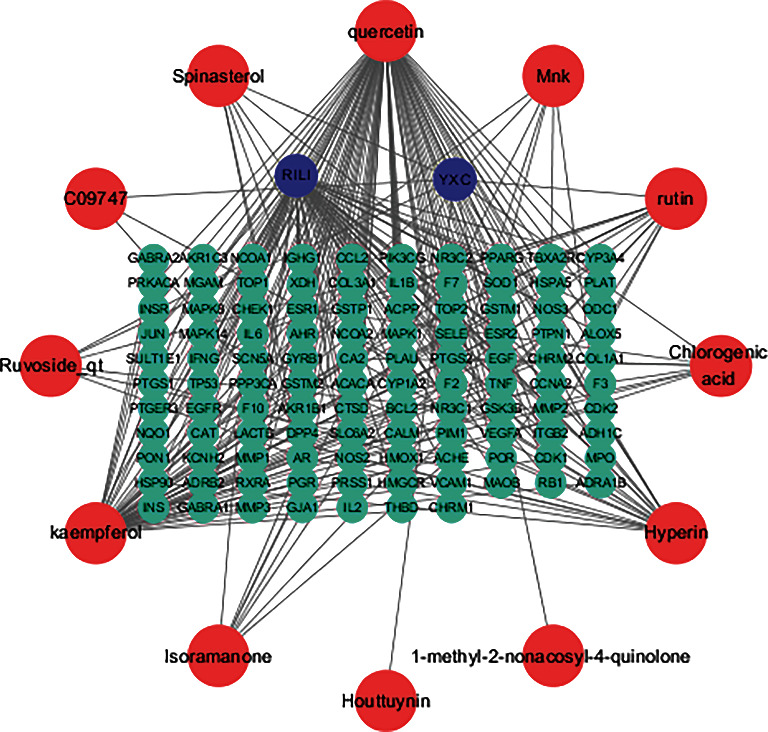
Topological network of drugs, chemical components, putative targets, and diseases. (The blue circles represent the disease and drug, the green circles represent the targets, the red circles represent the chemical components, and the gray lines between the two nodes represent the interaction).

As demonstrated in Figure 3, the “Drug-Compounds-Targets-Disease” network comprises 121 nodes and 107 common targets. Different targets potentially correspond to the same active compounds. Also, the same target can correspond to different active compounds. Each of the 12 active compounds interacts with at least 2 disease targets. Of note, *Houttuynia cordata* Thunb., used in the treatment of RILI has the characteristics of multiple components and multiple targets.

### 3.4. PPI Network and Hub Genes

**Figure 4:**
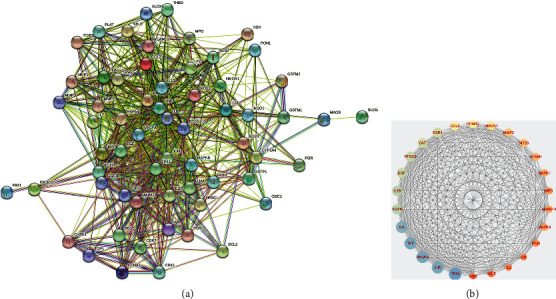
PPI network of overlapping genes. (a) PPI network of common genes. (b) Top 26 hub genes of 70 common genes by MCC algorithm. The larger the circle is, the greater the degree is.

The Intersection targets of “*Houttuynia cordata* Thunb.-RILI” were imported into the STRING database and used to plot the PPI network, with 62 nodes and 717 edges (Figure 4(a)). The PPI network was constructed via Cytoscape software for further visualization and analysis (Figure 4(b)). Results showed 26 key targets whose degrees were higher than or equal to 23.1 (the average score). The topological parameters of TOP10 Hub genes are shown in Table 2.

### 3.5. Gene Ontology and KEGG Enrichment Analysis

**Figure 5:**
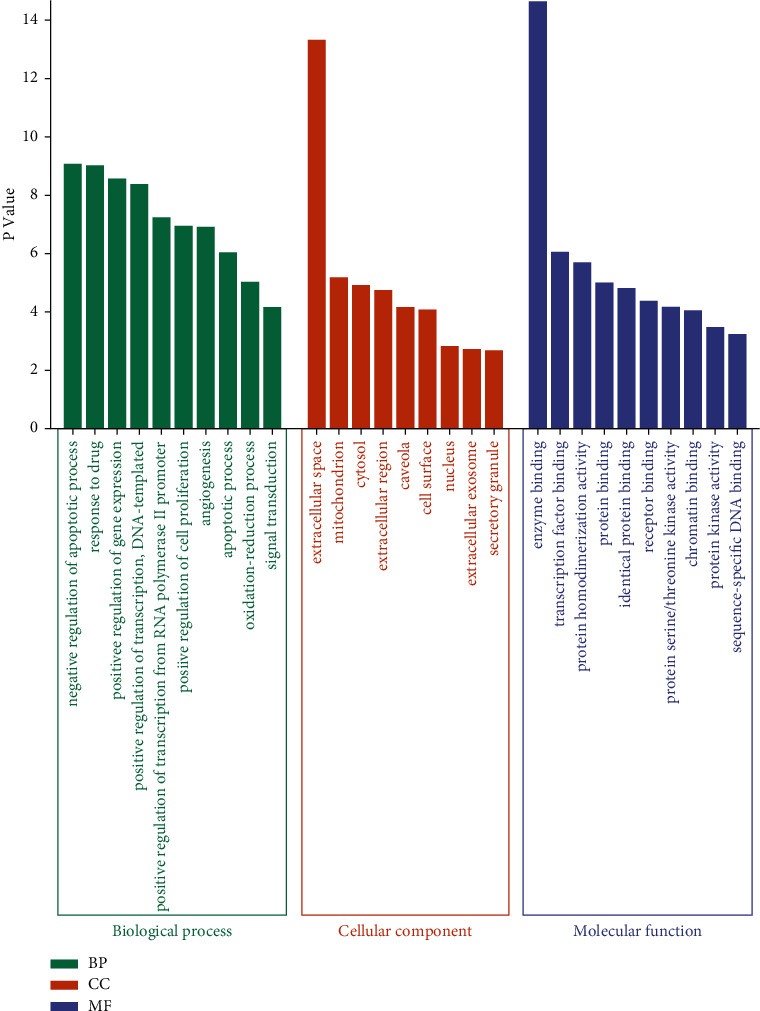
GO analysis of potential target genes of *Houttuynia cordata* Thunb. against RILI.

**Figure 6:**
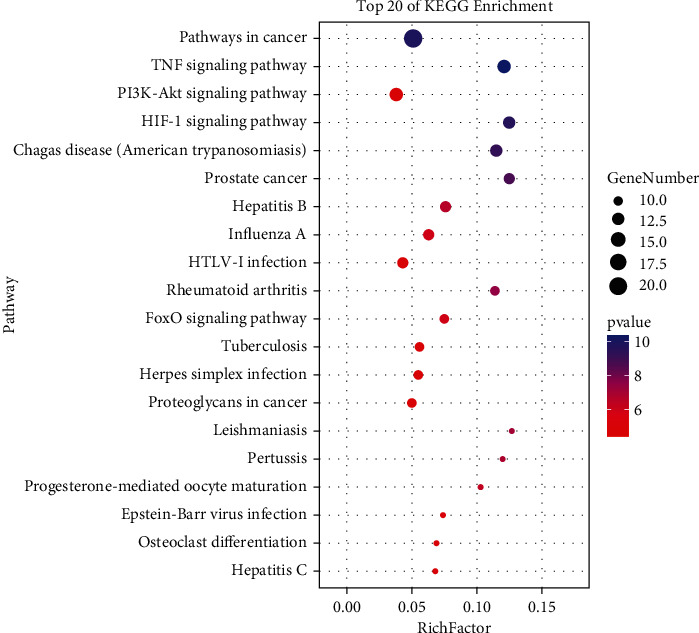
Pathway enrichment analysis of potential target genes of *Houttuynia cordata* Thunb. against RILI using the KEGG analysis.

**Figure 7:**
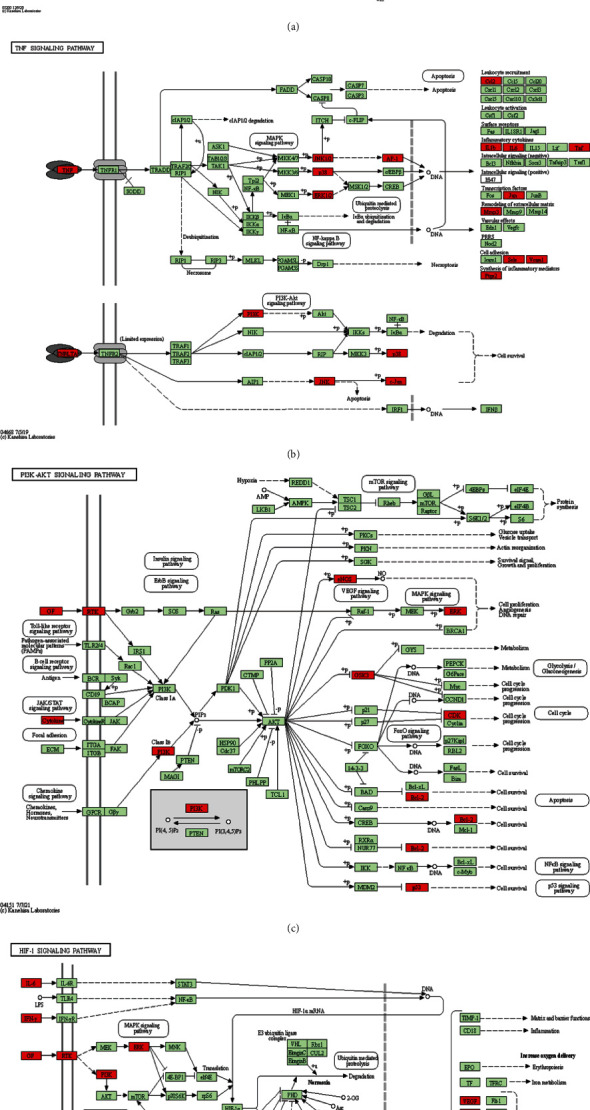
KEGG signaling pathways. (a) Cancer signaling pathway. (b) TNF signaling pathway. (c) PI3K-AKT signaling pathway. (d) HIF-1 signaling pathway.

The 62 common genes for drugs and diseases were uploaded to the DAVID website for GO and KEGG analysis. GO enrichment analysis yielded GO terms (*P* < 0.01, FDR < 0.05) comprising 112 biological processes (BP), 9 cellular components (CC), and 32 molecular functions (MF). The top 10 entries of BP, CC, and MF are displayed in Figure 5 in order of the lgP value, respectively. Additionally, 94 pathways were obtained via the KEGG pathway analysis. The top 20 entries were selected according to the lgP value to draw a bubble diagram (Figure 6). Analysis revealed the key genes were mainly enriched in the following pathways: cancer signaling pathway, TNF signaling pathway, PI3K-AKT signaling pathway, and HIF-1 signaling pathway (Figure 7).

### 3.6. Molecular Docking between Core Targets and Key Compounds

**Figure 8:**
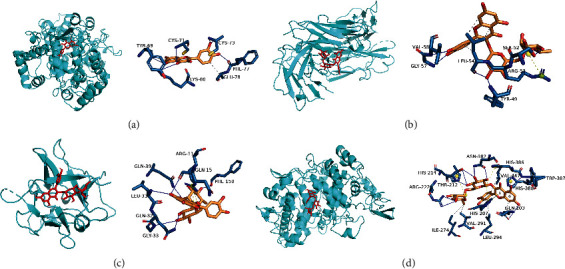
Simulated molecular docking of rutin on PTGS2, EGFR, IL-1B, and quercetin on PTGS2: (a) quercetin-PTGS2, (b) rutin-EGFR, (c) rutin-IL-1B, and (d) rutin-PTGS2.

The four key active components, quercetin, kaempferol, hyperin, and rutin, were used for molecular docking of 10 hub proteins, which are TP53 (PDB ID:4MZI), VEGFA (PDB ID:1KMX), JUN (PDB ID:1PXZ), TNF (PDB ID:4G4F), IL6 (PDB ID:1ALU), EGFR (PDB ID:2XKN), IL-1B (PDB ID:6Y8I), EGF (PDB ID:1EDM), PTGS2 (PDB ID:3NT1), and CAT (PDB ID:6GTK). Information about the affinity and hydrogen bond of compounds with key target protein is shown in Table 3. Binding energy less than 0 indicated that the ligand molecules could bind spontaneously to the receptor protein; binding energy less than −5.0 kcal/mol indicated good binding activity. Less than −5.0 kcal/mol binding energy was reported for effective active compounds of *Houttuynia cordata* Thunb. to the core target, demonstrating a good binding ability of them. Evidence on the combination of quercetin and PTGS2, rutin and PTGS2, EGFR, and IL-1B demonstrates the interaction between the active components (Figure 8).

## 4. Discussion

Compelling evidence shows that inflammation, oxidative stress, and fibrotic damage are the main pathological factors of RILI [30–32]. In view of the complicated pathogenesis, anti-RILI remains a considerable challenge in clinical practice, and current treatments are inadequate at present. Radiation is similar to the heat pathogen and toxin pathogen in TCM, which can injure the lung [33]. *Houttuynia cordata* Thunb. has upstanding clinical efficacy for RILI but the specific molecular mechanism of action is elusive. Recently, network pharmacology has become an advanced method to analyze the complex mechanisms and collect active components of Chinese herbs or TCM formulas. This study indicated that the complex network relationship of multicomponent, multitarget, and multipathway of *Houttuynia cordata* Thunb. in RILI treatment.

In the present study, we identified 12 active compounds of *Houttuynia cordata* Thunb. through network pharmacology. At the same time, 26 core targets of *Houttuynia cordata* Thunb. against RILI and 4 associated pathways were predicted. More evidence from this study indicates that flavonoids (quercetin, kaempferol, chrysin, and rutin), the main pharmacological active compounds of *Houttuynia cordata* Thunb., play a major inhibitory role against RILI. Flavonoids have previously been demonstrated to exert antiinflammatory, antioxidant, antitumor, antiradiation, and antifibrotic pharmacological effects [34]. In animal experimental studies, Quercetin was found to increase the content of Ikb-*α* protein content and decrease NF-kB protein level in the lung tissue of RILI mice. These events effectuated the regulation of the transcription of inflammatory factor genes, which successively improved the inflammatory exudation in mice with RILI and exerted a preventive effect on RILI [35]. In addition, to reduce the extent of RILI in mice, kaempferol blocks the expression of IL-6 and TNF-a, enhances total SOD activity, and inhibits NF-kB and MAPK pathways [36]. Rutin exerts the protective effect on lung injury via the following mechanisms: (i) block the release of inflammatory mediators by inhibiting the ERK/NB-kB signaling pathway activation; (ii) decrease the oxidative stress by increasing the activity of antioxidant enzymes, including CAT, GPx, and SOD [37, 38]. Moreover, molecular docking results verified that the core active compounds of *Houttuynia cordata* Thunb. show a good affinity to bind to the core targets. These data revealed potentially the main active components of *Houttuynia cordata* Thunb. against RILI, which provides a basis for clinical application.

Based on the PPI network, the core targets of *Houttuynia cordata* Thunb. in RILI treatment include TP53, JUN, TNF, IL-6, IL-1B, PTGS2, and CAT, which are both the key drug targets and the core targets of the disease, suggesting that these targets may be the direct targets of *Houttuynia cordata* Thunb. against RILI. Combined with GO enrichment and KEGG pathway analysis, the targets of *Houttuynia cordata* Thunb. in RILI treatment were mainly enriched in cancer pathways, TNF signaling pathways, PI3K-Akt signaling pathways, and HIF-1 signaling pathways, similar to previous reports in radiation pneumonia. In view of these findings, the potential mechanism of *Houttuynia cordata* Thunb. in RILI treatment is related to the following aspects: (1) cancer pathways are the most enriched pathways for *Houttuynia cordata* Thunb.-based RILI treatment, with TP53, JUN, and VEGF as tumor-related bases, which are the core targets. *Houttuynia cordata* Thunb. may act directly or indirectly on tumor-related targets to exert anti-RILI effects. Radiotherapy for thoracic tumors causes radiation lung injury, so antitumor therapy is potentially among the main therapeutic approaches for RILI. For instance, VEGF is the main inducer of vascular neovascularization, and evidence shows that radiotherapy-induced pulmonary fibrosis is accompanied by vascular neovascularization. VEGF can promote endothelial cell proliferation and increase vascular permeability, and this successively exacerbates the inflammatory response [39]. (2) Inflammation and fibrosis are crucial in the development and maintenance of RILI. Several targets of *Houttuynia cordata* Thunb. against RILI, among them, TNF, IL-6, and IL-1B, are mainly associated with the TNF signaling pathway. Therefore, the important anti-RILI mechanisms of *Houttuynia cordata* Thunb. are through antiinflammatory and antifibrotic effects. Of note, TNF is a key factor that initiates an inflammatory response *in vivo* and has been shown to play a critical role in early radiation pneumonia. Research using an animal model of pulmonary fibrosis also revealed that elevated synthesis and release of TNF stimulated the proliferation of fibroblasts and promoted collagen synthesis, events that caused pulmonary fibrosis. Inhibition of the TNF-a pathway demonstrated a significant protective effect on RILI in a mouse model of colon cancer lung metastasis [40]. Moreover, compelling evidence shows that TNF has a potentially critical role in the development of RILI by inducing oxidative stress through the release of reactive oxygen species. In this view, inhibition of TNF expression is suggested to be an effective strategy for RILI prevention and treatment [41, 42]. Studies have revealed that the expression levels of IL-6 and IL-1*β* a class of fibroblast growth factors, tend to increase after radiation and appear simultaneously with radiotherapy lung injury. Therefore, they are used frequently as a marker for the occurrence of lung injury after radiation therapy [43]. (3) PI3K/Akt signaling pathway is critical in the development of RILI. Regulation of the PI3K/Akt signaling pathway potentially inhibits collagen deposition, suppresses fibroblast activation, and attenuates radiation-induced lung fibrosis [44]. (4) HIF-1 is a typical hypoxia-inducible factor. Emerging evidence indicates a close association of hypoxia with radiation-induced inflammation, angiogenesis, and fibrosis [45]. Furthermore, PTGS2 and CAT are both oxidative stress-related enzymes, suggesting that *Houttuynia cordata* Thunb.-based prevention and treatment of RILI maybe through alleviating oxidative stress. Studies show that PTGS2 expression is positively correlated with the production of reactive oxygen species and inflammatory signals in tissues, and PTGS2 inhibition can reduce oxidative stress and inflammatory responses. The potential mechanism of the PTGS2 inhibitor, celecoxib, in RILI prevention and treatment is by reducing the toxicity of radiation to the lung [46, 47]. Taken together, the effects of the cancer pathway, TNF signaling pathway, PI3K-Akt signaling pathway, and HIF-1 signaling pathway are mainly related to inflammatory mediator synthesis, reduction of oxygen consumption, angiogenesis, tumor cell proliferation and apoptosis, DNA repair, etc. Therefore, it can be speculated that most of the active compounds of *Houttuynia cordata* Thunb. exert antitumor, antiinflammatory, antioxidative stress, antifibrosis effects and thus anti-RILI effects via the above pathways.

Collectively, the results in our study predict some potential therapeutic pathways and targets, and provide a reference for future research on *Houttuynia cordata* Thunb. treating RILI. However, a limitation is that further experiments are needed to demonstrate our findings.

## 5. Conclusion

In summary, this study preliminarily explored the active compounds, potential targets, and signaling pathways of *Houttuynia cordata* Thunb. for the treatment of RILI through network pharmacology and molecular docking. The results reflected the multicomponent, multitarget, and integrated regulation of TCM and provide a theoretical basis for the clinical application of *Houttuynia cordata* Thunb. on RILI treatment. However, this study did not consider the influence of drug dosage, drug ingredients, and drug delivery methods on the treatment results. Therefore, the results still need further verification (in vivo and in vitro) to provide the experimental basis for the future systematic study of *Houttuynia cordata* Thunb. on RILI treatment.

## Figures and Tables

**Table 1:** Effective active ingredients and parameters of *Houttuynia cordata* Thunb.

Herb	Mol ID	Molecule name	PubChem CID	OB (%)	DL
*Houttuynia cordata* Thunb.	MOL003851	Isochromanone	5318637	39.97	0.51
*Houttuynia cordata* Thunb.	MOL000422	Kaempferol	5280863	41.88	0.24
*Houttuynia cordata* Thunb.	MOL004345	1-methyl-2-nonacosyl-4-quinolone	5319734	31.54	0.50
*Houttuynia cordata* Thunb.	MOL004350	Ruvoside_qt	101650325	36.12	0.76
*Houttuynia cordata* Thunb.	MOL004351	C09747	442408	37.28	0.25
*Houttuynia cordata* Thunb.	MOL004355	Spinasterol	5281331	42.98	0.76
*Houttuynia cordata* Thunb.	MOL000098	Quercetin	5280343	46.43	0.28
*Houttuynia cordata* Thunb.	MOL004359	Houttuynia	122640	36.04	0.04
*Houttuynia cordata* Thunb.	MOL000924	Mnk	8163	17.66	0.03
*Houttuynia cordata* Thunb.	MOL000415	Rutin	5280805	3.20	0.68
*Houttuynia cordata* Thunb.	MOL003871	Chlorogenic acid	1794427	13.61	0.31
*Houttuynia cordata* Thunb.	MOL004368	Hyperin	5281643	6.94	0.77

**Table 2:** Hub genes in the PPI network (TOP10).

UniProt ID	Gene name	Protein name	Degree	Closeness centrality	Betweenness centrality
P04637	TP53	Cellular tumor antigen p53	49	0.82432432	0.05833188
P15692	VEGFA	Vascular endothelial growth factor A	47	0.80263158	0.04592475
P05412	JUN	Transcription factor AP-1	47	0.80263158	0.05446106
P01375	TNF	Tumor necrosis factor	47	0.80263158	0.04090074
P05231	IL6	Interleukin-6	46	0.79220779	0.03284284
P00533	EGFR	Epidermal growth factor receptor	41	0.74390244	0.06279942
P01584	IL1B	Interleukin-1 beta	41	0.74390244	0.01996984
P01133	EGF	Pro-epidermal growth factor	40	0.73493976	0.02295494
P35354	PTGS2	Prostaglandin G/H synthase 2	40	0.73493976	0.01653377
P04040	CAT	Catalase	39	0.73493976	0.07478328

**Table 3:** Molecular interactions of core targets and compounds (kcal/mol).

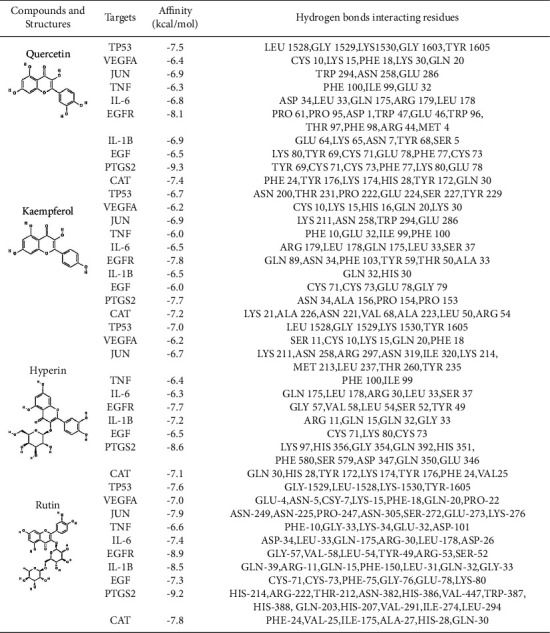

## Data Availability

All data are included in the manuscript and may be obtained from the corresponding authors via e-mail upon request.
